# Cerebral diffusion kurtosis imaging to assess the pathophysiology of postpartum depression

**DOI:** 10.1038/s41598-020-72310-1

**Published:** 2020-09-21

**Authors:** Yuri Sasaki, Kenji Ito, Kentaro Fukumoto, Hanae Kawamura, Rie Oyama, Makoto Sasaki, Tsukasa Baba

**Affiliations:** 1 Department of Obstetrics and Gynecology, Iwate Medical University School of Medicine, 2-1-1 Idaidori, Yahaba, Shiwa, Iwate 028-3695 Japan; 2 Division of Ultrahigh Field MRI, Institute for Biomedical Science, Iwate Medical University School of Medicine, Yahaba, Japan; 3 Department of Neuropsychiatry, Iwate Medical University School of Medicine, Yahaba, Japan

**Keywords:** Depression, Diagnostic markers, Diseases, Health care, Medical research, Pathogenesis

## Abstract

Postpartum depression (PPD), a main cause of maternal suicide, is an important issue in perinatal mental health. Recently, cerebral diffusion tensor imaging (DTI) studies have shown reduced fractional anisotropy (FA) in major depressive disorder (MDD) patients. There are, however, no reports using diffusion kurtosis imaging (DKI) for evaluation of PPD. This was a Japanese single-institutional prospective study from 2016 to 2019 to examine the pathophysiological changes in the brain of PPD patients using DKI. The DKI data from 3.0 T MRI of patients one month after delivery were analyzed; the patients were examined for PPD by a psychiatrist. The mean kurtosis (MK), FA and mean diffusivity (MD) were calculated from the DKI data and compared between PPD and non-PPD groups using tract-based spatial statistics analysis. Of the 75 patients analyzed, eight patients (10.7%) were diagnosed as having PPD. In the PPD group, FA values in the white matter and thalamus were significantly lower and MD values in the white matter and putamen were significantly higher. The area with significant differences in MD value was more extensive (40.8%) than the area with significant differences in FA value (6.5%). These findings may reflect pathophysiological differences of PPD compared with MDD.

## Introduction

According to a recent report, 60% of mothers who committed suicide in the first year of childbirth in the 23 wards of Tokyo had mental illness, and postpartum depression (PPD) was the most common (33%)^1^. The incidence of PPD has been reported to be 10–15%^2^ in developed countries and we reported an incidence of 11.3% in Japan in a previous study^3^. Depressive symptoms have been reported in more than 25% of perinatal women^2,4^. Forty cases of suicide within one year postpartum have been reported for the 10 years from 2005 to 2014 in Tokyo, 36% of which were PPD^1^. In addition, PPD has been reported to reduce mother-infant relationships including physical contact, laughter, and dialogue, and to affect the development of the child's cognition, emotions, motor functions, and nerve functions^5^. Thus, early detection of PPD and appropriate interventions are necessary. Currently, the most widely used PPD screening method is the Edinburgh Postnatal Depression Scale (EPDS) developed in 1987^6^. However, EPDS does not assess the pathophysiology of PPD.

Major depressive disorder (MDD) is one of the most prevalent psychiatric disorders. Several neuroimaging studies have suggested that abnormalities of the frontal-subcortical circuits in brain regions such as the lateral orbitofrontal, the dorsolateral prefrontal, and the anterior cingulate are involved in the pathology of MDD^7,8^. Based on these backgrounds, the relevance of neurocircuitry in PPD and differences between PDD and MDD are unknown.

Diffusion MRI is a widely used in-vivo imaging technique that measures the movement of water molecules in tissue, and recently, it has been used for clinical applications to diagnose neurodegenerative or psychiatric disorders^9,10^. Herein, we focused on an objective assessment of PPD using diffusion MRI. Diffusion-weighted imaging (DWI) and diffusion tensor imaging (DTI) are expressed using 1st order and 2nd order tensors, respectively^11^. Conventional DWI and DTI are inadequate to distinguish the actual diffusion because of the narrowing of the intercellular space and the complication of nerve fibers restricting the diffusion of water molecules in the central nervous system^12,13^. Therefore, as an advanced form of DTI, diffusion kurtosis imaging (DKI) using 4th-order tensor has been devised. DKI can sharply depict the structure of tissue by correcting the degree of restriction due to the structure of the tissue on the diffusion of water molecules as kurtosis^14,15^. This technique represents a promising approach for early diagnosis of various neurodegenerative diseases^16^. Although many studies have evaluated MDD using DTI ^17–30^, few studies have used DKI^31,32^. In those studies, the DKI showed a decrease in the mean kurtosis (MK) and radial kurtosis values, which were specific parameters that could not be expressed by DTI, in a wider range than that of fractional anisotropy (FA) value decreased. However, the pathophysiological mechanisms from these findings have not been elucidated. Moreover, the pathophysiological aspects of PPD are also poorly understood. In 2018, Silver et al. used DTI data to show that FA value declines in the brains of patients with PPD as well as MDD^33^. Although the clinical phenotype of PPD is similar to that of MDD, it is also suggested that PPD and MDD may not be the same^34^ and it is considered that the pathophysiology of PPD may be different from MDD. With this background in mind, we investigated the pathophysiological feature in the brain of patients with PPD and compared diffusion metrics between patients with and without PPD using DKI.

## Materials and methods

This study was conducted in compliance with the Declaration of Helsinki and according to the Ethical Guidelines for Medical and Health Research Involving Human Subjects established by the Ministry of Health, Labour, and Welfare in Japan. Written informed consent was obtained from all participants. The study protocol was approved by the ethical review committee of the Iwate Medical University Hospital (Approval No. H28-43). The protocol for this study was registered with UMIN Clinical Trials Registry (UMIN000037844).

### Patients

Eligible patients in the study were mothers who had delivered at the Iwate Medical University Hospital from August 2016 to February 2019, and were disqualified if they met any of the following exclusion criteria: 1) contraindications for MRI such as metal implants in the body or claustrophobia, 2) history of mental disorder including depression, 3) intracranial organic abnormality, 4) stillbirth delivery, 5) possibly be subjected to mental, physical or social burden by participating in the study, and 6) under 20 years old. Borderline cases and patients for whom it was difficult to determine whether or not they had PPD were excluded from analysis.

### Aim and procedure

The purpose of this study was to elucidate the pathophysiology of PPD by comparing the MK, FA, and MD values between patients with and without PPD. Eligible patients underwent an MRI at 1 month postpartum and a psychiatric assessment for PPD within 2 months postpartum (within 1 month of the MRI). According to the American Psychiatric Association, PPD was defined as having a primary symptom of MDD during pregnancy or within 4 weeks after delivery^35^, and the diagnostic criteria as listed in the Diagnostic and Statistical Manual of Mental Disorders, Fifth Edition, was used. Severity was classified as mild, moderate, and severe according to the diagnostic criteria.

### Imaging protocol

All subjects underwent an MRI examination with a 3-T scanner (Trillium Oval, Hitachi, Ltd., Tokyo, Japan). DKI source images were obtained using a single-shot spin-echo echo-planar-imaging sequence. Scanning parameters, set by referring to previous reports^36–41^, are shown in the Table 1. Interleaved acquisition was used to prevent the cross-talk artifact between adjacent slices, as described in previous studies^42,43^. Conventional MR images, including three-dimensional T1-weighted images and axial T2-weighted images, were also obtained.

**Table 1:** Acquisition parameters of diffusion MRI.

Parameter	Setting
Repetition time/echo time	4,500/110 ms
*b* values	0, 1,000, and 2,500 s/mm^2^
Motion probing gradients	20 directions
Matrix size	128 × 128 (256 × 256 after reconstruction)
Field of view	24 cm
Slice thickness	4.0 mm without inter-slice gaps
Number of excitations	4
Reduction factors of parallel imaging	2
Acquisition time	12 m 18 s

### Image analyses

Diffusion metric maps including MK, FA, and MD were calculated from the DKI source data using the in-house software program used in previous studies^36–41^. To identify changes in the whole-brain white matter between the PPD and non-PPD groups, we carried out voxel-wise statistical analysis of the MK, FA, and MD maps using tract-based spatial statistics (TBSS) implemented in Functional Magnetic Resonance Imaging of the Brain (FMRIB) Software Library 5.0.9^44,45^. Regarding the region of interest (ROI) analysis in the basal ganglia of the Johns Hopkins University Eve atlas^46^, MK, FA, and MD values of the caudate nucleus, putamen, globus pallidus, and thalamus were then automatically measured using coregistered ROIs^37,40,41^.

### Sample size

The ratio of patients with PPD to non-PPD was considered to be 1 to 8. Under the conditions of power 0.90, and α 0.05, the sample size required for the analysis was estimated to be 72 in total (8 cases of PPD and 64 cases of non-PPD). Assuming a dropout rate of 5% (4 cases), the number of enrolled patients was determined to be 76.

### Statistical analyses

For TBSS, to compare voxel values for MK, FA, and MD between patients with and without PPD, a two-sample t-test using non-parametric statistical inference function included in the FMRIB Software Library was performed with 5,000 permutation sets. All voxels in the brain were corrected using the threshold-free cluster enhancement method with the family-wise error correction. The ratios of the areas in the brain with significant changes in MK, FA, and MD to the mean FA skeleton area were calculated. A linear regression model was performed to investigate the relationship between MK, FA, or MD values and EPDS scores on TBSS. The Mann–Whitney U test was used for comparisons of MK, FA, and MD values obtained by ROI analysis between patients with and without PPD. We tested correlations between MK, FA, or MD values and EPDS scores on ROIs. For the clinical comparisons between the PPD and the non-PPD groups, a Mann–Whitney U test was used for continuous variables and a chi-square test was used for the categorical variables. For all statistical analyses, a significance level of *p* < 0.05 (two-sided) was used. Statistical analysis was performed using JMP ver. 13.1.0 software (SAS Institute Inc., Cary, NC, USA).

## Results

### Patient characteristics

**Figure 1:**
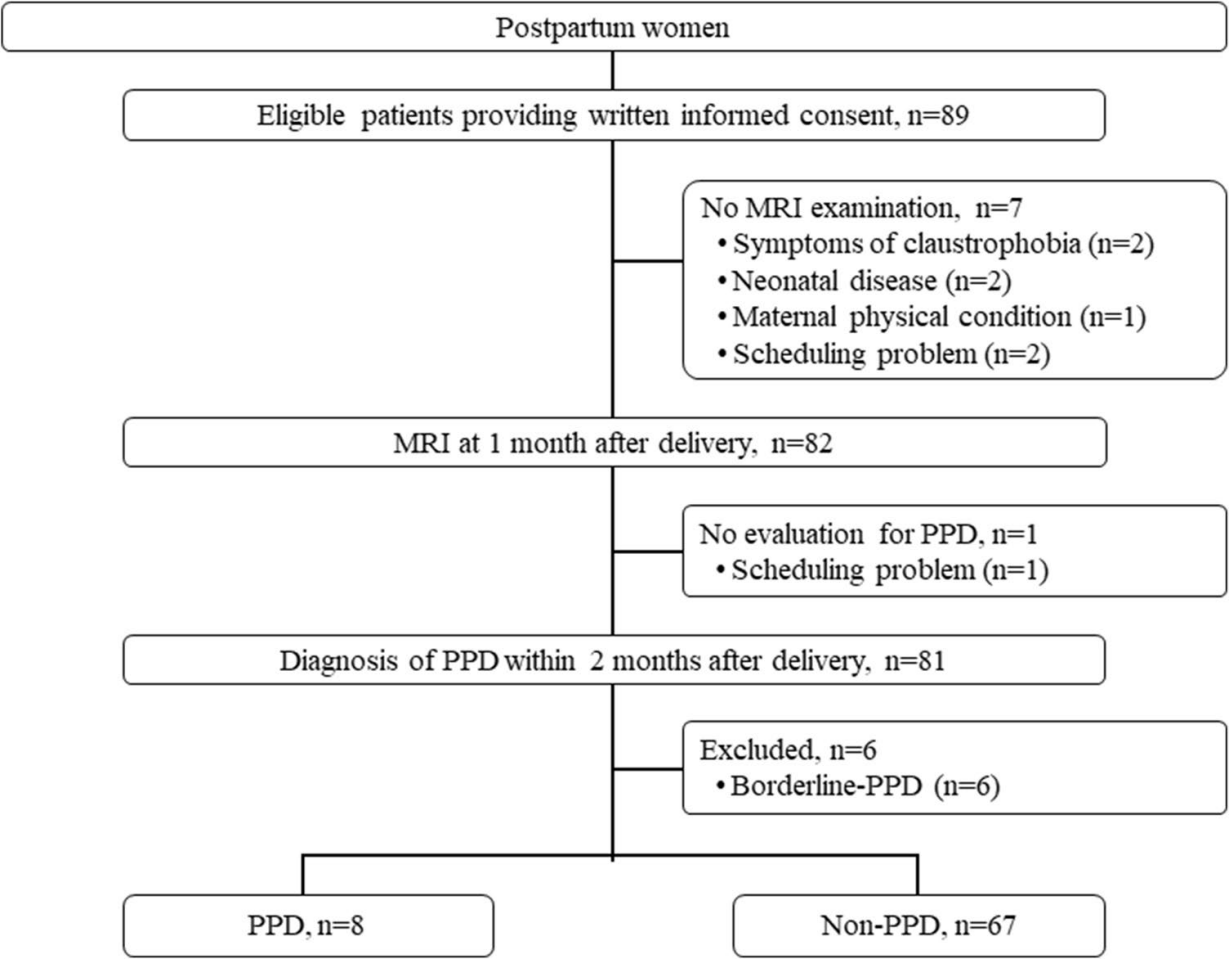
Patient flow. *PPD* postpartum depression.

Eighty-nine eligible patients were enrolled from August 2016 to March 2019 (Fig. 1). Seven patients were excluded because they had not undergone an MRI, and one patient was excluded because she had not undergone an examination for PPD. Among the 81 patients remaining, six patients were excluded because they were considered to be borderline cases; thus, 75 patients were analyzed. Eight patients (10.7%) were diagnosed as having PPD. Patient characteristics in the PPD and non-PPD groups are shown in Table 2. There were no significant differences in parity, maternal age, gestational age at delivery, birth weight, and Apgar scores between groups. There was a significant difference in the EPDS at one month after delivery. The individual background of the eight patients with PPD are shown in Table 3.

**Table 2:** PPD and non-PPD patient characteristics.

	PPD (n = 8)	Non-PPD (n = 67)	*p*
Age (years), mean ± SD	35.6 ± 6.3	35.1 ± 4.5	0.8027
Parity, n (%)			
Primipara	6 (75.0%)	43 (64.2%)	0.5433
Multipara	2 (25%)	24 (35.8%)	
Gestational age at delivery (weeks), mean ± SD	34.6 ± 6.1	37.3 ± 4.2	0.1890
Delivery route, n (%)			
Vaginal delivery	2 (25%)	26 (38.8%)	–
Selective Cesarean section	4 (50.0%)	17 (25.4%)	
Emergency Cesarean section	2 (25.0%)	24 (35.8%)	
1 M-EPDS, mean ± SD	11.9 ± 4.2	4.5 ± 3.3	0.0002
Birth weight, mean ± SD	2,146.8 ± 1,050.7	2,609.2 ± 750.8	0.2502
Apgar score, mean ± SD			
1 min	5.5 ± 3.5	7.0 ± 2.0	0.3628
5 min	7.5 ± 2.1	8.5 ± 1.2	0.2644
Admission to NICU of newborn infant, n (%)			
Yes	4 (50.0%)	18 (26.9%)	0.1743
No	4 (50.0%)	49 (73.1%)	

*PPD* postpartum depression, *1 M-EPDS* Edinburgh Postnatal Depression Scale at 1 month after delivery.

**Table 3:** Patient background.

Patient No	Age	Parity	Gestational week at delivery	Delivery	Birth weight (g)	Apgar score 1/5 min	NICU	1 M-EPDS	Clinical course and severity	1 M-MD		Follow-up MD	
										Cerebral white matter	Putamen	Cerebral white matter	Putamen
1	43	Primipara	40	Vaginal delivery (Induced labor)	3,358	8/9		13	Moderate until 1 month postpartum and mild at 2 months postpartum	0.7328	0.7246		
2	36	Primipara	36	Elective C/S	2,493	8/9		13	Mild until 1 month postpartum	0.7075	0.7248		
3	32	Multipara	37	Elective C/S	2,885	8/9		12	Onset after one month after delivery, mild	0.7314	0.7334	0.7178	0.6808
4	24	Primipara	32	Emergency C/S	1,424	1/5	Yes	5	Mild until 3 weeks after delivery	0.7177	0.7252		
5	35	Primipara	38	Vaginal delivery	3,279	9/9		20	Mild until 1 month after delivery	0.7029	0.69		
6	35	Primipara	39	Elective C/S	2,114	1/5	Yes	17	Mild until 1 month after delivery	0.7256	0.7071		
7	44	Multipara	23	Elective C/S+ATH	479	2/5	Yes	6	Mild until 2 weeks after delivery	0.7264	0.7278		
8	36	Multipara	28	Elective C/S	1,142	7/9	Yes	15	Mild until 1 month after delivery	0.6963	0.7079		

*1 M-EPDS* the Edinburgh Postnatal Depression Scale after 1 month of delivery, *1 M-MD* mean diffusivity value evaluated by MRI after 1 month of delivery, *C/S* cesarean section, *ATH* abdominal total hysterectomy.

### DKI analysis

**Figure 2:**
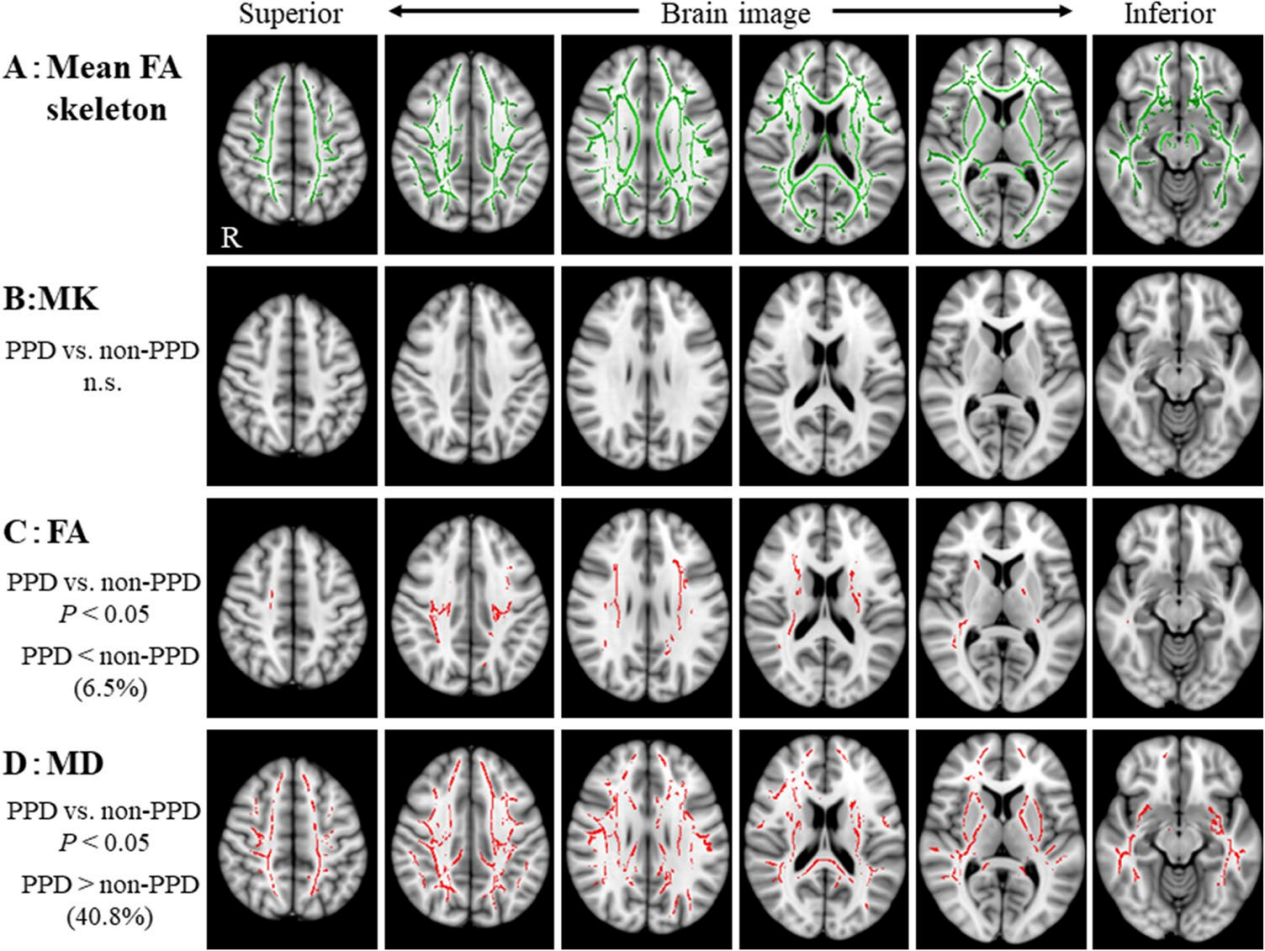
Tract-based spatial statistics (TBSS) show the areas of significant difference between the postpartum depression (PPD) group and the non-PPD group on six brain images from superior to inferior of diffusion kurtosis and tensor maps. A: Mean fractional anisotropy (FA) skeleton (shown in green) calculated for all subjects by TBSS. B: Mean kurtosis (MK) values were not significantly different between the PPD and the non-PPD groups. C: FA values were decreased in the PPD group compared with the non-PPD group. D: Mean diffusivity (MD) values were significantly increased in the PPD group compared with the non-PPD group. The areas with significantly decreased FA values or increased MD values in the PPD group are marked with red (P < 0.05, corrected for multiple comparisons). The statistical results are overlaid on the Montreal Neurological Institute 152-T1 standard brain template. The percentage in the left column represents the percentage of the significant voxels relative to the mean FA skeleton voxels for each parameter.

**Figure 3:**
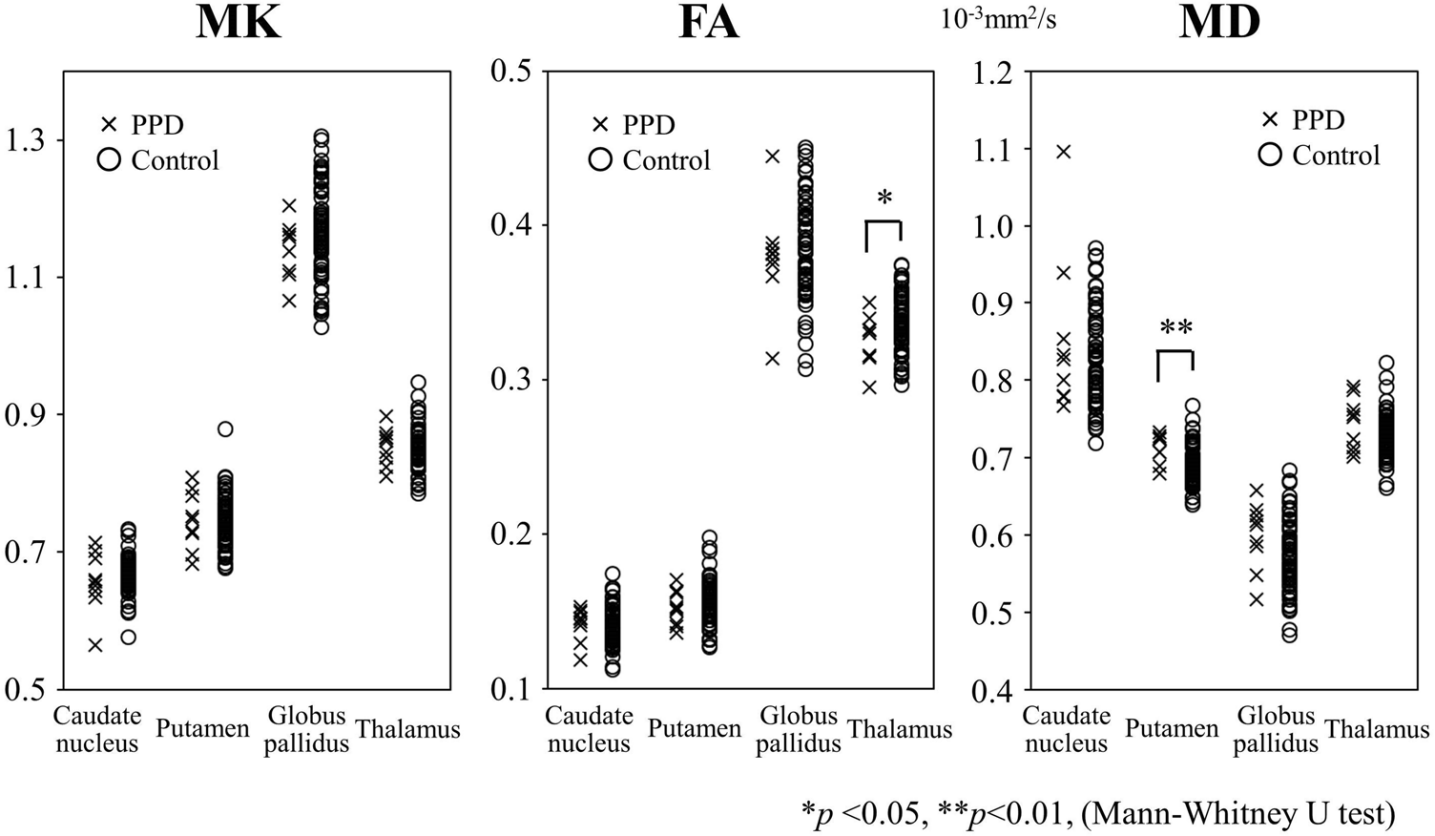
Diffusion kurtosis and tensor metrics of the patients with PPD and non-PPD. FA values of the thalamus were significantly decreased and MD values of the putamen were significantly increased in the PPD group compared with the non-PPD group. There are no significant differences in MK between the groups. *p < 0.05, **p < 0.01 (Mann–Whitney U test). Both MK and FA are unitless.

Figure 2 shows the results of the voxel-wise group analysis using TBSS. Compared with the non-PPD group, MD values for the PPD group were significantly higher in widespread white matter, including temporo-parietal regions, the superior longitudinal fasciculus, the corticospinal tract, the cingulum, the body/splenium of the corpus callosum, the external capsule, the anterior/posterior limb of the internal capsule, and the inferior longitudinal fasciculus, while FA values were significantly lower in the superior longitudinal fasciculus and the corticospinal tract. Moreover, the rate for areas in the brain with significant changes in FA and MD voxels relative to the mean FA skeleton voxels were 6.5% and 40.8%, respectively. There were no significant differences in MK values in white matter between groups. Regarding the ROI analysis, FA values of the thalamus in the PPD group were significantly lower (median [range]: 0.32 [0.30–0.35]) than those of the non-PPD group (0.34 [0.30–0.38]) (*p* = 0.047) and MD values of the putamen in the PPD group were significantly higher (0.73 [0.69–0.73]) than those of the non-PPD group (0.69 [0.64–0.77]) (*p* = 0.003) (Fig. 3). There were no significant differences in MK values between groups in the caudate nucleus, putamen, globus pallidus, and thalamus (MK, *p* = 0.79–0.18). Additionally, there were no significant correlations across groups between MK, FA, or MD values and EPDS scores on TBSS or ROIs.

### Severity and course of PPD

Seven of the eight patients (87.5%) were diagnosed as having mild PPD (Table 3 and Supplemental Fig. 1). At one month postpartum, when MRI examination was performed, the symptoms of PPD were over peak in most patients (Table 3 and Supplmental Fig. 2). One patient (Patient No.3 in Table 3) who had developed symptoms after 1 month postpartum had improved at 7 months postpartum. An additional MRI at 10 months after delivery in the patient showed a tendency of decrease in the MD values of white matter and putamen that had shown an increase in the first MRI examination 1 month after delivery (Supplemental Fig. 2).

## Discussion

We investigated structural changes in the brain in patients with PPD using DKI. Our major findings were that patients with PPD showed a significant decrease in FA value, a significant increase in MD value, and no significant difference in MK value compared with non-PPD patients. Most interesting, significant differences in MD values were found in a wider area than in areas where FA values were significantly different.

We found decreased FA in the PPD group within the superior longitudinal fasciculus and the corticospinal tract using TBSS analysis and the thalamus using ROI analysis. A recent study showed no significant differences in FA between the PPD and non-PPD groups using TBSS, while they revealed significantly reduced FA values of the left anterior limb of the internal capsule using ROI^47^. However, we detected no significant difference in the structure using ROI (data not shown).

Many studies of MDD have shown significant differences in FA between MDD and non-MDD patients^17,18,20,25,28,29^. Recently, the largest multi-site DTI study in MDD showed evidence for global differences in FA and radial diffusivity in multiple WM, including the corpus callosum, internal capsule, corona radiata, cingulum, and fornix, in MDD^48^. Global lower FA in some WM tracts in MDD was accompanied by global higher radial diffusivity, yet not differences found in MD. Although our results showed significant differences in FA and MD values between the PDD and non-PDD groups, the rate for the area with significant changes in MD voxels relative to the mean FA skeleton voxels (40.8%) was higher than that of FA (6.4%). The increase in MD value may reflect the pathophysiological characteristics of PPD, while decrease in FA value may reflect those of MDD. In this study, most PPD patients had mild PPD; however, if PPD becomes chronic or transitions to MDD, the rate of change in FA may further increase. In order to prove that change in MD value reflects a PPD characteristic, further studies are necessary that compare MD values before, during, and after pregnancy.

**Figure 4:**
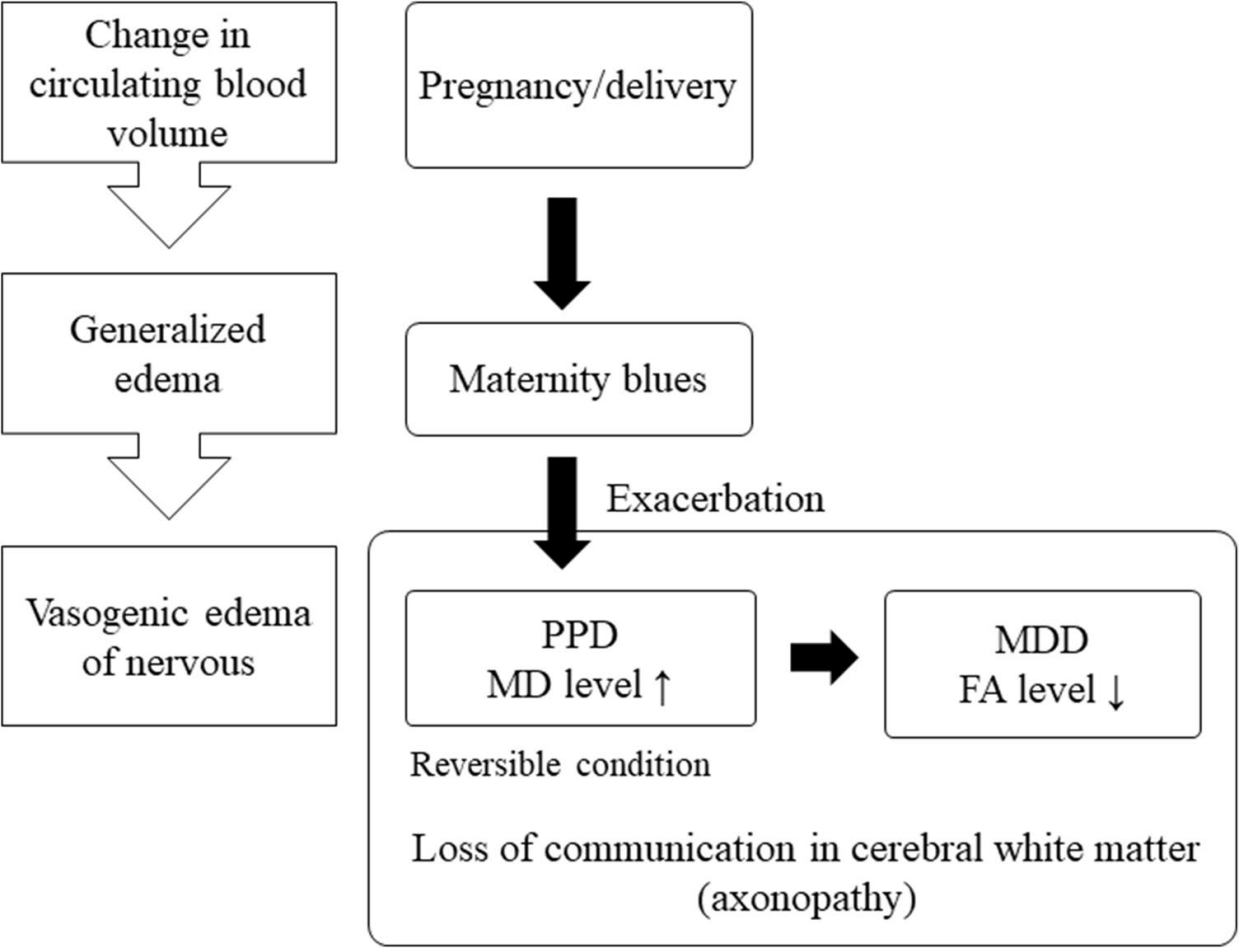
Hypothetical mechanism of PPD. FA: fractional anisotropy, MD: mean diffusivity, MMD: major depressive disorder, PPD: postpartum depression.

In general, an increase in MD is thought to be associated with elevated extracellular water content^49^. FA is believed to reflect more evident destruction of tissue architecture, such as axonal degeneration and demyelination^50^, whereas MK is considered to reflect subtle pathological changes including microgliosis, reactive astrogliosis, or decreased myelin, axonal or neuronal density^51–53^. Therefore, we hypothesized that an increase in MD value reflects neurogenic vasogenic edema associated with changes in circulating blood volume due to pregnancy and parturition (Fig. 4). An increase in circulating blood volume due to pregnancy may cause edema in not only systemic but also in neural tissue. Maternity blues or PPD as clinical phenotypes may develop depending on the severity of the neural tissue edema and both conditions are considered to be reversible^54^. This presumed mechanism is consistent with PPD being a more reversible disease than MDD. Generally, increased radial diffusivity reflects mainly demyelination^55,56^. Therefore, MDD may be associated with widespread destruction of tissue architecture, and widespread structural dysconnectivity may play a role in the pathophysiology of MDD.

In this study, we did not detect any significant correlations across groups between MK, FA or MD values and EPDS scores on TBSS or ROIs analyses. A previous study showed a negative correlation between FA and EPDS scores in the left anterior limb of the internal capsule, in the right retrolenticular internal capsule, and the body of the corpus callosum^33^. This difference may be due to the different patient characteristics. Future studies should further examine this.

The strength of this study is that the pathophysiology of PPD was objectively evaluated using DKI technology. Another strength is the previously unreported finding of a significant increase in the MD value in patients with PPD.

On the other hand, our study also has the following limitations: 1) this is a single-site study and generalizations from the results are not guaranteed, 2) it was difficult to obtain informed consent from patients with unstable mental status, and this might cause selection bias, 3) there is a limit of power depending on the number of patients, and 4) most patients were diagnosed as having mild PPD.

Another issue is that the choice of acquisition DKI parameters could affect the evaluation of the diffusion weighted data. First, we used a minimum echo time (110 ms), which was comparable to that of previous DTI studies in patients with MDD on 1.5 T^20^. However, the value was longer that of previous studies on 3 T^20^, because we used high *b*-values of 2,500 s/mm^2^ for the DKI protocol. Hence, we applied four signal averages to maintain sufficient signal-to-noise ratio. Second, the lowest *b*-values was set to 0 s/mm^2^. At *b*-values lower than 200 s/mm^2^, blood perfusion might affect the measurement signal magnitude, thereby biasing the results^57^. Third, due to its dense vascular network, the diffusion metrics in the basal ganglia might be somewhat affected by this effect. Although we applied four signal averages to reduce the effects of cardiac pulsations, such as signal loss or signal attenuation, other acquisition techniques, such as cardiac gating, may be needed for further accuracies of the diffusion metrics^58^. Forth, a set of 20 diffusion gradient directions with four signal averages was chosen. Although accuracies of diffusion metrics are known to depend on the number of directions^59^, a previous study revealed that DTI data using 6 directions with five or ten averages are comparable to those using 30 or 60 directions with one average^60^. Therefore, the DTI metrics we obtained are roughly comparable to those that have 80 directions. In addition, MK values we obtained are corresponding to those derived from the optimized scan parameters^36^. Finally, relatively thick sections, i.e., 4 mm, should be used to maintain an adequate signal-to-noise ratio even on images with high *b*-values of 2,500 s/mm^2^. Therefore, the DKI metrics in this study may be include errors due to partial volume effects^61^, which may deteriorate the accuracy of our results.

Our study suggests that PPD strongly affects MD in large sections of the brain, but caused only small changes in FA and MK. To the best of our knowledge, this is a first study that applied recently developed DKI method to PPD. Hence, we need to test the reproducibility of diffusion metrics across different scanners or different field of strengths. In addition, to prove the pathophysiology of PPD further, brain imaging studies that compare MK, FA, and MD values before, during and after pregnancy are needed. Therefore, further studies are needed to clarify whether measuring the MK is valuable on PPD considering the high *b*-values and long acquisition times required by the DKI method, and a future prospective longitudinal study using high-resolution images at 3 T is planned.

Although further studies are warranted, our findings exhibit the potency of diffusion MRI for not only early detection but assessment of the PPD recovering process. Furthermore, by elucidating the neurogenic vasogenic edematous status throughout a pregnancy using MRI, a novel therapeutic approach for PPD, such as anti-edema drugs, may result. We hope that the findings herein will contribute to the development of better PPD treatment.

## Supplementary information

Supplementary information
