# Supplementary material for: Cerebral diffusion kurtosis imaging to assess the pathophysiology of postpartum depression
Source: Sci Rep. 2020 Sep 21;10:15391. doi: 10.1038/s41598-020-72310-1 (PMC7505968; doi:10.1038/s41598-020-72310-1)

Cerebral diffusion kurtosis imaging to assess the pathophysiology of postpartum depression

Yuri Sasaki[1*], Kenji Ito[2], Kentaro Fukumoto[3], Hanae Kawamura[1], Rie Oyama[1], Makoto Sasaki[2], and Tsukasa Baba[1]

[1]Department of Obstetrics and Gynecology, Iwate Medical University School of Medicine, Yahaba, Japan
[2]Division of Ultrahigh Field MRI, Institute for Biomedical Science, Iwate Medical University School of Medicine, Yahaba, Japan
[3]Department of Neuropsychiatry, Iwate Medical University School of Medicine, Yahaba, Japan

**Correspondence should be addressed to Y.S. (email: yurisasa@iwate-med.ac.jp)*

Supplemental Figure 1

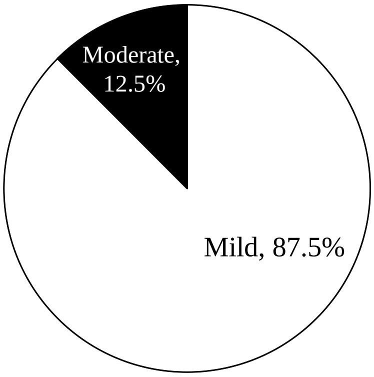

A. Severity (n=8)

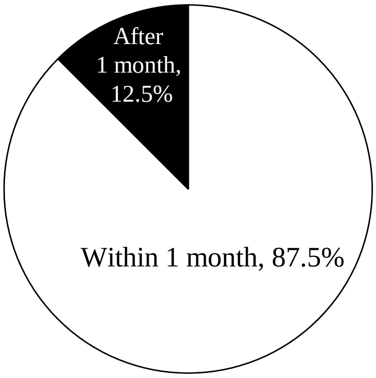

B. Peak time of symptoms (n=8)

Supplemental Figure 2

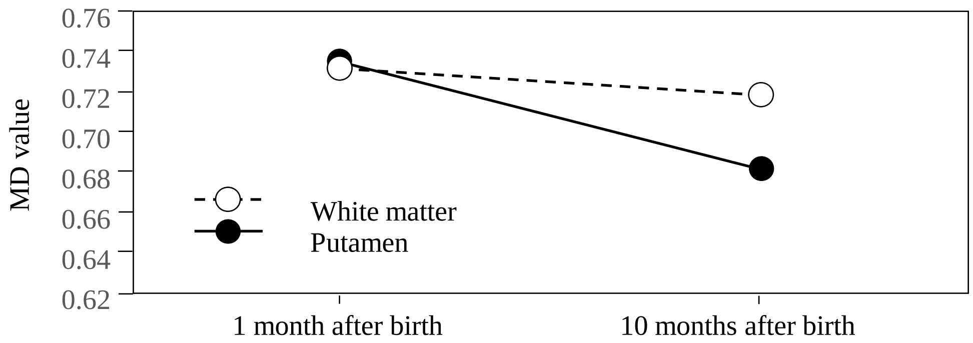

Supplement: Supplementary file 1 — Supplementary information [file 41598_2020_72310_MOESM1_ESM.pdf]
